# Infrared Image Super-Resolution Reconstruction Based on Quaternion and High-Order Overlapping Group Sparse Total Variation

**DOI:** 10.3390/s19235139

**Published:** 2019-11-23

**Authors:** Xingguo Liu, Yingpin Chen, Zhenming Peng, Juan Wu

**Affiliations:** 1 School of Information and Communication Engineering, University of Electronic Science and Technology of China, Chengdu 611731, China; xgliu9066@163.com; 2 Laboratory of Imaging Detection and Intelligent Perception, University of Electronic Science and Technology of China, Chengdu 610054, China; 3 Chongqing College of Electronic Engineering, Chongqing 401331, China; juanwucq@126.com; 4 School of Physics and Information Engineering, Minnan Normal University, Zhangzhou 363000, China; 110500617@163.com

**Keywords:** infrared image, super-resolution, overlapping group sparse, regularization by denoising, high-order gradient

## Abstract

Owing to the limitations of imaging principles and system imaging characteristics, infrared images generally have some shortcomings, such as low resolution, insufficient details, and blurred edges. Therefore, it is of practical significance to improve the quality of infrared images. To make full use of the information on adjacent points, preserve the image structure, and avoid staircase artifacts, this paper proposes a super-resolution reconstruction method for infrared images based on quaternion total variation and high-order overlapping group sparse. The method uses a quaternion total variation method to utilize the correlation between adjacent points to improve image anti-noise ability and reconstruction effect. It uses the sparsity of a higher-order gradient to reconstruct a clear image structure and restore smooth changes. In addition, we performed regularization by using the denoising method, alternating direction method of multipliers, and fast Fourier transform theory to improve the efficiency and robustness of our method. Our experimental results show that this method has excellent performance in objective evaluation and subjective visual effects.

## 1. Introduction

Image super-resolution reconstruction (SRR) uses digital signal processing to generate high-resolution (HR) images from a single or multiple frames of low-resolution (LR) images, mainly through the super-resolution method. Image super-resolution reconstruction can efficiently utilize the potential value of existing image data and has applications such as military remote sensing reconnaissance [1], target tracking and monitoring [2,3,4], target location and recognition [5], astronomical observation [6], and medical imaging [7].

There are three types of super-resolution reconstruction methods: based on regular terms representation, learning-based methods, and partial differential equation-based methods. Learning-based image super-resolution reconstruction has been studied extensively in the recent years. For example, based on the convolutional neural network (CNN), Lim proposed an enhanced deep super-resolution network (EDSR) by removing unnecessary modules [8]. Dong redesigned the super-resolution CNN (SRCNN) structure by introducing a deconvolution layer at the end of the network, reformulating the mapping layer, adopting smaller filter sizes [9]. Xu proposed a novel global dense feature fusion convolutional network (DFFNet), which can take full advantage of global intermediate features leading to a continuous global information memory mechanism [10]. To restore various scales of image details, Du enhanced the multi-scale inference capability of CNNs by introducing competition among multi-scale convolutional filters [11]. Chi proposed a uniform deep CNN (DCNN) framework to handle the denoising and super-resolution of the CT image at the same time [12]. Zhang made a comparative study of fast super-resolution CNN (FSRCNN), deeply recursive convolutional networks (DRCN), very deep super-resolution convolutional networks (VDSR) and SRCNN for single image super-resolution with the purpose of space applications, and concluded that DRCN is the best model with more generalized for space object image [13]. Xiao formulated a joint loss function by combining the output and high-dimensional features of a non-linear mapping network, which uses satellite video data itself as a training set [14]. For infrared images, Liu proposed a classified dictionary learning method which classifies features of the samples into several reasonable clusters and trained a dictionary pair for each cluster [15]. He proposed a cascaded architecture of deep neural networks with multiple receptive fields by a large scale factor (×8) [16]. These methods learn the mapping between HR and LR images by pre-selecting test samples and accordingly reconstruct HR images. They can achieve good reconstruction results; however, the computational complexity is high.

The image reconstruction method based on the partial differential equation model has good results. The most popular of these methods are those based on the total variation (TV) regularization model [17]. This method preserves the edges of the images well, while removing image noise. However, there are “staircase artifacts” and unclear texture problems in the reconstructed image. To reduce the staircase artifacts, some scholars have proposed high-order variational models [18,19]. For example, Bredies, Kunisch, and Pock proposed total generalized variation (TGV) based on the combination of TV regularization with higher-order derivatives [20]. Although these methods can reduce staircase artifacts and protect the edges of the image, they produce “spots effect” in the processed image. To balance staircase artifacts and spot effect, a fractional-order variational model, which uses a fractional gradient instead of an integer gradient, has been proposed [21,22,23]. We have also proposed a super-resolution method, which combines quaternion [24,25] and fractional-order total variation, and uses the ADMM acceleration algorithm, achieving good results in image objective evaluation, visual effect and duration [26].

The regular term representation is an image representation model that captures the main information and intrinsic geometry of the image with a few parameters and achieves good results in terms of image restoration, target tracking, and other applications. Since Yang et al. first applied sparse representation to super-resolution reconstruction [27,28], many scholars have proposed improved methods for super-resolution reconstruction based on sparse representation [29,30,31,32,33,34,35]. In recent years, Selesnick and Chen proposed overlapping group sparse total variation (OGSTV) [36], which is a non-separating regular term that preserves the sparsity of the objective function [37]. The overlapping group sparse regularization term considers the sparsity of the image difference domain and also mines the neighborhood difference information of each point, thus mining structural sparsity characteristics of the image gradient. By overlapping the combined gradients, the difference between the smooth region and the boundary region can be improved, thereby suppressing the staircase artifacts of the TV model. The work of Selesnick and Chen, Liu et al. generalized the one-dimensional overlapping sparse regularization term into a two-dimensional overlapping sparse regularization term and introduced it into an anisotropic total variational model for denoising and deconvolution [38,39,40]. Using the Lp quasinorm instead of the L1 norm, we have also proposed a method for infrared image deblurring with an overlapping group sparse total variation method, in which the Lp quasinorm introduces another degree of freedom, better describes image sparsity characteristics, and improves image restoration [41].

Besides, there are some other types of image reconstruction models. Wang proposed an image self-embedding method, using authentication watermark and recovery watermark to complete image restoration. The authentication watermark locates the tampered area. The recovery watermark is compressed into different categories and encoded into variable lengths to improve the quality of the recovered images [42]. Xia proposed a new fast and accurate image matching algorithm, which first presents the district-identification method to obtain the integer-pixel matching result, then introduce gradient algorithm to match the sub-pixel position [43]. Wang proposed an image authentication and a recovery algorithm based on chaos and Hamming code, which can effectively detect image tampering and complete image recovery [44]. Wang proposed an image tampering detection and recovery algorithm based on jitter and chaos technology. The algorithm uses chaos technology to complete watermark embedding and encryption. Combined with the Chinese remainder theorem, it further reduces the impact of watermark embedding on image quality [45].

In fact, for the noisy images, the conventional super-resolution way is to denoise the images as a pre-processing step and then super-resolve the denoised images. In some new methods [46,47,48,49], such as the median filter transform (MFT) with parallelogram-shaped windows [47], denoising and super-resolving are integrated to provide improved results in comparison to the conventional way.

Super-resolution models based on regular terms can be solved by the alternating direction method of multipliers (ADMM) algorithm [50]. In recent years, many scholars have proposed various algorithms based on the classic ADMM, such as the plug-and-play (PnP) ADMM [51,52,53,54,55] and and regularization by denoising (RED) framework [56,57,58,59]. They are powerful image-recovery frameworks that aim to minimize an explicit regularization objective constructed from a plug-in image-denoising function. Since their introduction, they have demonstrated extremely promising results in image restoration and signal recovery problems [60,61,62].

In this study, we explore quaternion total variation and high-order to improve the sparsity exploitation of OGSTV. Our proposed method is called the quaternion and high-order overlapping group sparse (HOGS4), which is efficiently solved through the RED framework. The novelty of our work is two-fold. First, the HOGS4 method is considerably less restrictive than the OGSTV method for infrared image reconstruction as it shows good performance in terms of detail preservation by incorporating high-order image derivatives and also achieves accurate measurement of the sparsity potential from prior regularity. Second, it provides fast and efficient closed-form solutions for computationally complex sub-minimization problems using FFT.

The remainder of this paper is organized as follows. Section 2 briefly introduces the majorization–minimization (MM) method and RED framework. Section 3 describes the proposed method. In Section 4, our experiments and results are described. Finally, Section 5 and Section 6 present the discussion and conclusions, respectively.

## 2. Related Works

### 2.1. Overlapping Group Sparse Total Variation

The overlapping group sparse total variation (OGSTV) model [36] is as follows:

(1) $$R_{\mathrm{OGSTV}}\left(F\right)=\varphi \left(K_{1}*F\right)+\varphi \left(K_{2}*F\right),$$

where the symbol * is the convolution operator; $F\in R^{N\times N}$ is the reconstructed image; $K_{1}=\left[-1,1\right]$ and $K_{2}=\left[\begin{array}{c} -1\\ 1 \end{array}\right]$ are the horizontal and vertical differential convolution kernels, respectively. $\varphi \left(V\right)=\sum_{i=1}^{N}\sum_{j=1}^{N}{∥{\tilde{V}}_{i,j,K,K}∥}_{2}$ is used to solve the combined gradient, where ${\tilde{V}}_{i,j,K,K}$ is defined as

(2) $${\tilde{V}}_{i,j,K,K}=\left[\begin{array}{cccc} V_{i-K_{l},j-K_{l}} & V_{i-K_{l},j-K_{l}+1} & \cdots & V_{i-K_{l},j+K_{r}}\\ V_{i-K_{l}+1,j-K_{l}} & V_{i-K_{l}+1,j-K_{l}+1} & \cdots & V_{i-K_{l}+1,j+K_{r}}\\ \vdots & \vdots & \ddots & \vdots \\ V_{i+K_{r},j-K_{l}} & V_{i+K_{r},j-K_{l}+1} & \cdots & V_{i+K_{r},j+K_{r}} \end{array}\right],$$

where K is the group size, $K_{l}=\left\lfloor \frac{K-1}{2}\right\rfloor$, $K_{r}=\left\lfloor \frac{K}{2}\right\rfloor$. ⌊x⌋ is the largest integer value less than or equal to x.

From Equation (2), it can be seen that the combined gradient considers the gradient information of the neighborhood pixel, and the gradient information of these neighboring pixels is recombined by the L2 norm, thereby improving the difference between the smooth region and the edge region of the image [39].

The overlapping group sparse model can be solved using the MM method [63]:

(3) $$P\left(V\right)=prox_{\gamma \varphi }\left(V_{0}\right)=\underset{V}{\arg\min}\frac{1}{2}{∥V-V_{0}∥}_{2}^{2}+\gamma \varphi \left(V\right),$$

where $\varphi \left(V\right)$ is the overlapping group sparse regular term, and ${\tilde{V}}_{i,j,K,K}$ is an overlapping group sparse matrix of size K×K.

According to the MM method, to minimize $P\left(V\right)$, we need to find a function $Q\left(V,U\right)$, such that $Q\left(V,U\right)\geq P\left(V\right)$ for all V and U, and the equality holds if and only if V=U. According to this, the minimum value of $Q\left(V,U\right)$ calculated each time is the optimized value of $P\left(V\right)$, and Equation (3) can be written as

(4) $$V^{\left(k+1\right)}=\underset{V}{\arg\min}Q\left(V,V^{\left(k\right)}\right).$$

According to the following inequalities:

(5) $$\frac{1}{2}\left(\frac{1}{{∥U∥}_{2}}{∥V∥}_{2}^{2}+{∥U∥}_{2}\right)\geq {∥V∥}_{2},$$

where the equal sign is only true when U=V.

From Equations (3) and (5), we can obtain the optimization terms of $\varphi \left(V\right)=\sum_{i=1}^{N}\sum_{j=1}^{N}{∥{\tilde{V}}_{i,j,K,K}∥}_{2}$ as shown below:

(6) $$S\left(V,U\right)=\frac{1}{2}\sum_{i=1}^{N}\sum_{j=1}^{N}\left(\frac{1}{{∥U_{i,j,K,K}∥}_{2}}{∥V_{i,j,K,K}∥}_{2}^{2}+{∥U_{i,j,K,K}∥}_{2}\right)\geq \varphi \left(V\right)=\sum_{i=1}^{N}\sum_{j=1}^{N}{∥{\tilde{V}}_{i,j,K,K}∥}_{2}$$

Equation (6) can be written as:

(7) $$S\left(V,U\right)=\frac{1}{2}{∥D\left(U\right)v∥}_{2}^{2}+C\left(U\right),$$

where v is the vector form of the matrix V, $C\left(U\right)$ is independent of V and can be considered as a constant term for V; $D\left(U\right)$ is a diagonal matrix whose diagonal elements are defined as follows:

(8) $${\left[D\left(U\right)\right]}_{m,m}=\sqrt{\sum_{i=-K_{l}}^{K_{r}}\sum_{j=-K_{l}}^{K_{r}}{\left\{\sum_{k_{1}=-K_{l}}^{K_{r}}\sum_{k_{2}=-K_{l}}^{K_{r}}{\left|U_{m-i+k_{1},m-j+k_{2}}\right|}^{2}\right\}}^{-\frac{1}{2}}}.$$

By combining Equations (4) and (6), Equation (3) can be transformed into the following iterative optimization method:

(9) $$\begin{array}{cc} V^{\left(k+1\right)} & =\underset{V}{\arg\min}\frac{1}{2}{∥V-V_{0}∥}_{2}^{2}+\gamma S\left(V,V^{\left(k\right)}\right)\\ \; & =\underset{V}{\arg\min}\frac{1}{2}{∥V-V_{0}∥}_{2}^{2}+\gamma \left\{\frac{1}{2}{∥D\left(V^{\left(k\right)}\right)v∥}_{2}^{2}+C\left(V^{\left(k\right)}\right)\right\}. \end{array}$$

Its iterative optimal solution is as follows:

(10) $$V^{\left(k+1\right)}=\mathrm{mat}\left\{{\left(I+\gamma D^{2}\left(V^{\left(k\right)}\right)\right)}^{-1}v_{0}\right\},$$

where I is the identity matrix, $v_{0}$ is the vector form of $V_{0}$, and mat represents the vector matrixing operator.

Therefore, we obtain Algorithm 1 to solve Equation (3).

**Algorithm 1** MM method**Initialize:**$V=V_{0}$, γ, $K^{2}$, $K_{l}=\left\lfloor \frac{K-1}{2}\right\rfloor$, $K_{r}=\left\lfloor \frac{K}{2}\right\rfloor$, ε, Maximum inner iterations NIt, n=0 **While**$\frac{{∥V^{\left(n+1\right)}-V^{\left(n\right)}∥}_{2}}{{∥V^{\left(n\right)}∥}_{2}}\;\;>\;\;\varepsilon \;\;or\;\;n<\mathrm{NIt}$**do** compute ${\left[D^{2}\left(V^{\left(n\right)}\right)\right]}_{m,m}=\sum_{i=-K_{l}}^{K_{r}}{\sum_{j=-K_{l}}^{K_{r}}\left\{\sum_{k_{1}=-K_{l}}^{K_{r}}\sum_{k_{2}=-K_{l}}^{K_{r}}{\left|V_{m-i+k_{1},m-j+k_{2}}^{\left(k\right)}\right|}^{2}\right\}}^{-\frac{1}{2}}$compute $V^{\left(n+1\right)}=\mathrm{mat}\left\{{\left(I+\gamma D^{2}\left(V^{\left(n\right)}\right)\right)}^{-1}V_{0}\right\}$compute n=n+1 **End While** **Return**$V^{\left(n\right)}$

### 2.2. Regularization by Denoising

For image super-resolution reconstruction, the model can be expressed as

(11) $$F=\underset{F}{\arg\min}\frac{1}{2}{∥\mathrm{SHF}-G∥}_{2}^{2}+\mu R\left(F\right),$$

where H is a circular matrix that represents the convolution for the anti-aliasing filter. S is a binary sampling matrix, where the rows are subsets of the identity matrix. Further, G is an observation image, and F represents the corresponding original image.

To solve the above model, we can transform it into image denoising using regularization by denoising (RED) [56,57], which relies on a general structural smoothness penalty term for regularizing any desired inverse problem. Specifically, the regularization term $R\left(F\right)$ is defined as

(12) $$R\left(F\right)=\frac{1}{2}F^{T}\left(F-f\left(F\right)\right),$$

where $f\left(F\right)$ is defined as the image denoising engine

(13) $$f\left(F\right)=\underset{\hat{F}}{\arg\min}\frac{1}{2}{∥\hat{F}-F∥}_{2}^{2}+\lambda \psi \left(\hat{F}\right).$$

The denoising engine is applied to image F, and the induced penalty is proportional to the inner product between the image and its denoising residual. The smooth regularization effectively uses image adaptive Laplacian, and then extracts its definition from any image denoising engine f(·). Interestingly, under the mild assumption of f(·), it is proved that the regularized gradient is manageable, just like the given denoising residual F−f(F) [58,59].

## 3. Proposed Method

Inspired by the overlapping group sparse and quaternion total variation methods, this paper proposed a denoising model that uses the RED framework to complete infrared image super-resolution reconstruction (HOGS4). The traditional OGSTV does not fully consider pixel points, only considers first-order information [40]. To improve the denoising effect, we extend the traditional OGSTV to the high-order total variation model. The proposed model of high-order overlapping group sparse total variation not only considers first-order information but also adds the high-order gradient information of the horizontal, vertical, back diagonal and diagonal directions to the prior term. The introduction of quaternion and high-order information is used to make the prior knowledge more accurate, thus protecting the edges of the image [26], and also suppressing the influence of small edges on the estimation of the blurring core [20]. The denoising model is defined as follows:

(14) $$f_{\mathrm{HOGS}4}\left(F\right)=\underset{F}{\arg\min}\frac{1}{2}{∥F-G∥}_{2}^{2}+\sum_{i=1}^{4}\left(\lambda_{i}\varphi \left(K_{i}*F\right)+\omega_{i}{∥K_{i}*K_{i}*F∥}_{2}^{2}\right),$$

where $K_{i}\left(i=1,2,3,4\right)$ represents the convolution kernels along the horizontal, vertical, back diagonal, and diagonal directions, respectively. These are defined as follows:

(15) $$K_{1}=\left[-1,1\right],K_{2}=\left[\begin{array}{c} -1\\ 1 \end{array}\right],K_{3}=\left[\begin{array}{cc} 0 & -1\\ 1 & 0 \end{array}\right],K_{4}=\left[\begin{array}{cc} -1 & 0\\ 0 & 1 \end{array}\right].$$

Then according to Equation (11), the HOGS4 for infrared image super-resolution reconstruction method based on RED framework can be expressed as:

(16) $$F=\underset{F}{\arg\min}\frac{1}{2}{∥\mathrm{SHF}-G∥}_{2}^{2}+\mu R_{\mathrm{HOGS}4}\left(F\right),$$

where regularization term $R_{\mathrm{HOGS}4}\left(F\right)$ in RED framework can be defined as

(17) $$R_{\mathrm{HOGS}4}\left(F\right)=\frac{1}{2}F^{T}\left(F-f_{\mathrm{HOGS}4}\left(F\right)\right).$$

To solve the HOGS4 model in the RED framework, according to the principle of ADMM, an assistant variable Z is required to convert the unconstrained problem given by Equation (16) into a constrained problem:

(18) $$\left(F,Z\right)=\underset{F,Z}{\arg\min}\frac{1}{2}{∥\mathrm{SHF}-G∥}_{2}^{2}+\mu R_{\mathrm{HOGS}4}\left(Z\right),s.t\;\;Z=F.$$

Consequently, the corresponding augmented Lagrangian function is as follows:

(19) $$L\left(F,Z,Y\right)=\frac{1}{2}{∥\mathrm{SHF}-G∥}_{2}^{2}+\mu R_{\mathrm{HOGS}4}\left(Z\right)+\frac{\rho }{2}{∥F-Z+Y∥}_{2}^{2},$$

where Y is a Lagrange multiplier, and  ρ>0 is a penalty parameter.

Because F and Z are decoupled, the minimizer of Equation (18) can be found by solving the following sequence of F and Z sub-problems:

(20) $$F^{\left(k+1\right)}=\underset{F}{\arg\min}\frac{1}{2}{∥\mathrm{SHF}-G∥}_{2}^{2}+\frac{\rho }{2}{∥F-Z^{\left(k\right)}+Y^{\left(k\right)}∥}_{2}^{2},$$

(21) $$Z^{\left(k+1\right)}=\underset{Z}{\arg\min}\mu R_{\mathrm{HOGS}4}\left(Z\right)+\frac{\rho }{2}{∥F^{\left(k+1\right)}-Z+Y^{\left(k\right)}∥}_{2}^{2}.$$

The procedure comprises the following steps:

1. To solve the sub-problem of F, let W=SH. Then, Equation (20) can be represented as follows:

(22) $$F^{\left(k+1\right)}=\underset{F}{\arg\min}\frac{1}{2}{∥\mathrm{WF}-G∥}_{2}^{2}+\frac{\rho }{2}{∥F-Z^{\left(k\right)}+Y^{\left(k\right)}∥}_{2}^{2}.$$

Considering $Z^{\left(k\right)}$ and $Y^{\left(k\right)}$ are fixed, by setting the first-order derivative of F in Equation (22) as zero, we have

(23) $$0=W^{T}\left(\mathrm{WF}-G\right)+\rho \left(F-Z^{\left(k\right)}+Y^{\left(k\right)}\right).$$

According to the ADMM, the solution of the sub-problem of F is

(24) $$F^{\left(k+1\right)}={\left[W^{T}W+\rho I\right]}^{-1}\left[W^{T}G+\rho \left(Z^{\left(k\right)}-Y^{\left(k\right)}\right)\right]$$

2. To solve the sub-problem Z, according to Equations (17) and (21) can be transformed as follows:

(25) $$Z^{\left(k+1\right)}=\underset{Z}{\arg\min}\frac{\mu }{2}Z^{T}\left(Z-f_{\mathrm{HOGS}4}\left(Z\right)\right)+\frac{\rho }{2}{∥F^{\left(k+1\right)}-Z+Y^{\left(k\right)}∥}_{2}^{2}.$$

Considering $F^{\left(k+1\right)}$ and $Y^{\left(k\right)}$ are fixed, by setting the first-order derivative of Z in Equation (25) as zero, we have

(26) $$0=\mu \left(Z-f_{\mathrm{HOGS}4}\left(Z\right)\right)+\rho \left(Z-F^{\left(k+1\right)}-Y^{\left(k\right)}\right),$$

which can be solved by the fixed point strategy, leading to the following update rule for Z [56]:

(27) $$Z^{\left(j+1\right)}=\frac{1}{\mu +\rho }\left(\mu f_{\mathrm{HOGS}4}\left(Z^{\left(j\right)}\right)+\rho \left(F^{\left(k+1\right)}+Y^{\left(k\right)}\right)\right),$$

where $f_{\mathrm{HOGS}4}\left(\cdot \right)$ is HOGS4 denoising engine, which is defined as:

(28) $$f_{\mathrm{HOGS}4}\left(Z^{\left(j\right)}\right)=\underset{\hat{Z}}{\arg\min}\frac{1}{2}{∥\hat{Z}-Z^{\left(j\right)}∥}_{2}^{2}+\sum_{i=1}^{4}\left(\lambda_{i}\varphi \left(K_{i}*\hat{Z}\right)+\omega_{i}{∥K_{i}*K_{i}*\hat{Z}∥}_{2}^{2}\right).$$

Euqation (27) means that our approach in this case is computationally more expensive, as it will require several activations of the denoising engine $f_{\mathrm{HOGS}4}$ [56].

3. Then we update the Lagrange multiplier as

(29) $$Y^{\left(k+1\right)}=Y^{\left(k\right)}+\gamma \rho \left(F^{\left(k+1\right)}-Z^{\left(k+1\right)}\right).$$

The proposed SRR method is summarized in Algorithm 2.

**Algorithm 2** Super-resolution using RED-HOGS4**Initialize:**ρ, μ, N **While**${∥\mathrm{PSNR}\left(F^{\left(k+1\right)},G\right)-\mathrm{PSNR}\left(F^{\left(k\right)},G\right)∥}_{2}^{2}>tol$ compute $F^{\left(k+1\right)}=\underset{F}{\arg\min}\frac{1}{2}{∥\mathrm{WF}-G∥}_{2}^{2}+\frac{\rho }{2}{∥F-Z^{\left(k\right)}+Y^{\left(k\right)}∥}_{2}^{2}$compute ${\tilde{Z}}^{\left(1\right)}=Z^{\left(k\right)}$forj=1,2,…,Ncompute ${\hat{Z}}^{\left(j\right)}=f_{\mathrm{HOGS}4}\left({\tilde{Z}}^{\left(j\right)}\right)$compute ${\tilde{Z}}^{\left(j+1\right)}=\frac{\mu }{\mu +\rho }{\hat{Z}}^{\left(j\right)}+\frac{\rho }{\mu +\rho }\left(F^{\left(k+1\right)}+Y^{\left(k\right)}\right)$endforcompute $Z^{\left(k+1\right)}={\tilde{Z}}^{\left(N\right)}$compute $Y^{\left(k+1\right)}=Y^{\left(k\right)}+\gamma \rho \left(F^{\left(k+1\right)}-Z^{\left(k+1\right)}\right)$ **End While**

Regarding the sub-problem Z, Equation (28) can be converted into the following constraint problem:

(30) $$\begin{array}{c} \hat{Z}=\underset{\hat{Z}}{\arg\min}\frac{1}{2}{∥\hat{Z}-Z^{\left(j\right)}∥}_{2}^{2}+\sum_{i=1}^{4}\left(\begin{array}{c} \lambda_{i}\varphi \left(V_{i}\right)+\omega_{i}{∥W_{i}∥}_{2}^{2} \end{array}\right),\\ s.t.V_{i}=K_{i}*\hat{Z},W_{i}=K_{i}*K_{i}*\hat{Z}\;\;\left(i=1,2,3,4\right). \end{array}$$

Accordingly, the augmented Lagrangian function is:

(31) $$\begin{array}{cc} L\left(\hat{Z},V_{i},W_{i};U_{vi},U_{wi}\right) & =\frac{1}{2}{∥\hat{Z}-Z^{\left(j\right)}∥}_{2}^{2}\\ \; & +\sum_{i=1}^{4}\left(\lambda_{i}\varphi \left(V_{i}\right)+\omega_{i}{∥W_{i}∥}_{2}^{2}\right)\\ \; & +\sum_{i=1}^{4}\frac{\eta_{vi}}{2}{∥V_{i}-K_{i}*\hat{Z}-U_{vi}∥}_{\begin{array}{c} 2 \end{array}}^{2}\\ \; & +\sum_{i=1}^{4}\frac{\eta_{wi}}{2}{∥W-K_{i}*K_{i}*\hat{Z}-U_{wi}∥}_{2}^{2}, \end{array}$$

where $U_{vi}$ and $U_{wi}$ (i=1,2,3,4) is the Lagrange multipliers; $\eta_{vi}>0$ and $\eta_{wi}>0$ are penalty parameters.

The minimizer of Equation (30) is the saddle point of $L\left(\hat{Z},V_{i},W_{i};U_{vi},U_{wi}\right)$, which can be found by solving the following sequence of subproblems:

(32) $$\begin{array}{cc} {\hat{Z}}^{\left(n+1\right)} & =\underset{\hat{Z}}{\arg\min}\frac{1}{2}{∥\hat{Z}-Z^{\left(j\right)}∥}_{2}^{2}+\sum_{i=1}^{4}\frac{\eta_{vi}}{2}{∥V_{i}^{\left(n\right)}-K_{i}*\hat{Z}-U_{vi}^{\left(n\right)}∥}_{2}^{2}\\ \; & +\sum_{i=1}^{4}\frac{\eta_{wi}}{2}{∥W_{i}^{\left(n\right)}-K_{i}*K_{i}*\hat{Z}-U_{wi}^{\left(n\right)}∥}_{2}^{2} \end{array}$$

(33) $$\begin{array}{c} V_{i}^{\left(n+1\right)}=\underset{V_{i}}{\arg\min}\lambda_{i}\varphi \left(V_{i}\right)+\frac{\eta_{vi}}{2}{∥V_{i}-\left(K_{i}*{\hat{Z}}^{\left(n+1\right)}+U_{vi}^{\left(n\right)}\right)∥}_{2}^{2},\left(i=1,2,3,4\right) \end{array}$$

(34) $$\begin{array}{c} W_{i}^{\left(n+1\right)}=\underset{W_{i}}{\arg\min}\omega_{i}{∥W_{i}∥}_{2}^{2}+\frac{\eta_{wi}}{2}{∥W_{i}-\left(K_{i}*K_{i}*{\hat{Z}}^{\left(n+1\right)}+U_{wi}^{\left(n\right)}\right)∥}_{2}^{2},\left(i=1,2,3,4\right) \end{array}$$

The procedure comprises the following steps:

1. To solve the sub-problem $\hat{Z}$, the 2D Fourier transform of $\hat{Z}$ can be obtained by employing the convolution theorem [64]:

(35) $$\begin{array}{cc} F\left({\hat{Z}}^{\left(n+1\right)}\right) & =\underset{\hat{Z}}{\arg\min}\frac{1}{2}{∥F\left(\hat{Z}\right)-F\left(Z^{\left(j\right)}\right)∥}_{2}^{2}\\ \; & +\sum_{i=1}^{4}\frac{\eta_{vi}}{2}{∥F\left(V_{i}^{\left(n\right)}\right)-F\left(K_{i}\right)\circ F\left(\hat{Z}\right)-F\left(U_{vi}^{\left(n\right)}\right)∥}_{2}^{2}\\ \; & +\sum_{i=1}^{4}\frac{\eta_{wi}}{2}{∥F\left(W_{i}^{\left(n\right)}\right)-F\left(K_{i}\right)\circ F\left(K_{i}\right)\circ F\left(\hat{Z}\right)-F\left(U_{wi}^{\left(n\right)}\right)∥}_{2}^{2} \end{array}$$

where the symbol ∘ represents component-wise multiplication.

Considering $Z^{\left(j\right)}$, $V_{i}^{\left(n\right)}$, $W_{i}^{\left(n\right)}$, $U_{vi}^{\left(n\right)}$ and $U_{wi}^{\left(n\right)}$ are fixed, by setting the first-order derivative of $\hat{Z}$ in Equation (35) as zero, we have   

(36) $$\begin{array}{cc} 0 & =F\left(\hat{Z}\right)-F\left(Z^{\left(j\right)}\right)\\ \; & -\sum_{i=1}^{4}\left\{\eta_{vi}{\left[F\left(K_{i}\right)\right]}^{*}\circ \left[F\left(V_{i}^{\left(n\right)}\right)-F\left(K_{i}\right)\circ F\left(\hat{Z}\right)-F\left(U_{vi}^{\left(n\right)}\right)\right]\right\}\\ \; & -\sum_{i=1}^{4}\left\{\eta_{wi}{\left[F\left(K_{i}\right)\circ F\left(K_{i}\right)\right]}^{*}\circ \left[F\left(W_{i}^{\left(n\right)}\right)-F\left(K_{i}\right)\circ F\left(K_{i}\right)\circ F\left(\hat{Z}\right)-F\left(U_{wi}^{\left(n\right)}\right)\right]\right\}. \end{array}$$

For simplicity, we abbreviate Equation (36) as

(37) $$\begin{array}{c} F\left(\hat{Z}\right)\circ \mathrm{lhs}=\mathrm{rhs}, \end{array}$$

where

(38) $$\begin{array}{cc} \mathrm{lhs} & =I+\sum_{i=1}^{4}\left\{\eta_{vi}{\left[F\left(K_{i}\right)\right]}^{*}\circ F\left(K_{i}\right)\right\}\\ \; & +\sum_{i=1}^{4}\left\{\eta_{wi}{\left[F\left(K_{i}\right)\circ F\left(K_{i}\right)\right]}^{*}\circ \left[F\left(K_{i}\right)\circ F\left(K_{i}\right)\right]\right\} \end{array}$$

(39) $$\begin{array}{cc} \mathrm{rhs} & =F\left(Z^{\left(j\right)}\right)+\sum_{i=1}^{4}\left\{\begin{array}{c} \eta_{vi}{\left[F\left(K_{i}\right)\right]}^{*}\circ \left[F\left(V_{i}^{\left(n\right)}\right)-F\left(U_{vi}^{\left(n\right)}\right)\right] \end{array}\right\}\\ \; & +\sum_{i=1}^{4}\left\{\begin{array}{c} \eta_{wi}{\left[F\left(K_{i}\right)\circ F\left(K_{i}\right)\right]}^{*}\circ \left[F\left(W_{i}^{\left(n\right)}\right)-F\left(U_{wi}^{\left(n\right)}\right)\right] \end{array}\right\} \end{array}$$

Then, according to Equation (37), we have

(40) $$\begin{array}{c} {\hat{Z}}^{\left(n+1\right)}=F^{-1}\left(\mathrm{rhs}\cdot /\mathrm{lhs}\right) \end{array}$$

where ${\left[F\left(K_{i}\right)\right]}^{*}$ is the conjugate map of $F\left(K_{i}\right)$.

2. To solve the sub-problem of $V_{i}$ in Equation (33), the MM (Algorithm 1) can be used:

(41) $$\begin{array}{c} V_{i\left(m+1\right)}^{\left(n+1\right)}=\mathrm{mat}\left\{{\left(I+\frac{\lambda_{i}}{\eta_{vi}}D^{2}\left(V_{i\left(m\right)}^{\left(n+1\right)}\right)\right)}^{-1}V_{i\left(0\right)}^{\left(n+1\right)}\right\},\left(i=1,2,3,4\right) \end{array}$$

where $V_{i\left(m+1\right)}^{\left(n+1\right)}$ represents the iteration of the MM algorithm for $V_{i}^{\left(n+1\right)}$, and $V_{i\left(0\right)}^{\left(n+1\right)}$ as

(42) $$\begin{array}{c} V_{i\left(0\right)}^{\left(n+1\right)}=K_{i}*{\hat{Z}}^{\left(n+1\right)}+U_{vi}^{\left(n\right)} \end{array}$$

3. To solve the sub-problem $W_{i}$, we set the first-order derivative of $W_{i}$ in Equation (34) as zero, and get:

(43) $$\begin{array}{c} W_{i}^{\left(n+1\right)}=F^{-1}\frac{\eta_{wi}\left[F\left(K_{i}\right)\circ F\left(K_{i}\right)\circ F\left({\hat{Z}}^{\left(n+1\right)}\right)+F\left(U_{wi}^{\left(n\right)}\right)\right]}{\left(2\omega_{i}+\eta_{wi}\right)I},\left(i=1,2,3,4\right) \end{array}$$

4. Lastly, the Lagrange multiplier can be updated as

(44) $$\begin{array}{c} \left\{\begin{array}{c} U_{vi}^{\left(n+1\right)}=U_{vi}^{\left(n\right)}-\gamma \eta_{vi}\left(V_{i}^{\left(n+1\right)}-K_{i}*{\hat{Z}}^{\left(n+1\right)}\right)\\ U_{wi}^{\left(n+1\right)}=U_{wi}^{\left(n\right)}-\gamma \eta_{wi}\left(W_{i}^{\left(n+1\right)}-K_{i}*K_{i}*{\hat{Z}}^{\left(n+1\right)}\right) \end{array}\right.,\left(i=1,2,3,4\right) \end{array}$$

In this manner, all the sub-problems of Equation (28) are solved independently. In all iterations, the sub-problem V is solved by MM algorithm according to Equations (41) and (42). Considering the special structure of the differential matrices in the sub-problem of W, we regard the differential operators as convolution operators. By introducing the convolution theorem [64], the sub-problem W is solved in the frequency domain. The entire algorithm to solve Equation (28) is summarized in Algorithm 3. Besides, for Algorithm 3, regarded as HOGS4 denoising engine, we can also use it as an independent denoising algorithm, using quaternion and high-order overlapping group sparse total variation to complete the image denoising.

**Algorithm 3** HOGS4 denoising engine using ADMM
**Initialize:**

**While**
${\left\|\mathrm{PSNR}\left({\hat{Z}}^{\left(i+1\right)},Z^{\left(j\right)}\right)-\mathrm{PSNR}\left({\hat{Z}}^{\left(i\right)},Z^{\left(j\right)}\right)\right\|}_{2}^{2}>tol$

compute ${\hat{Z}}^{\left(i+1\right)}$ according to Equations (38)–(40);compute $V^{\left(i+1\right)}$ according to Equations (41) and (42);compute $W^{\left(i+1\right)}$ according to Equation (43);compute $U_{vi}$ and $U_{wi}$ according to Equation (44);

**End While**

**Return**
${\hat{Z}}^{\left(i+1\right)}$


## 4. Experiments and Results

### 4.1. Materials and Method

**Figure 1:**
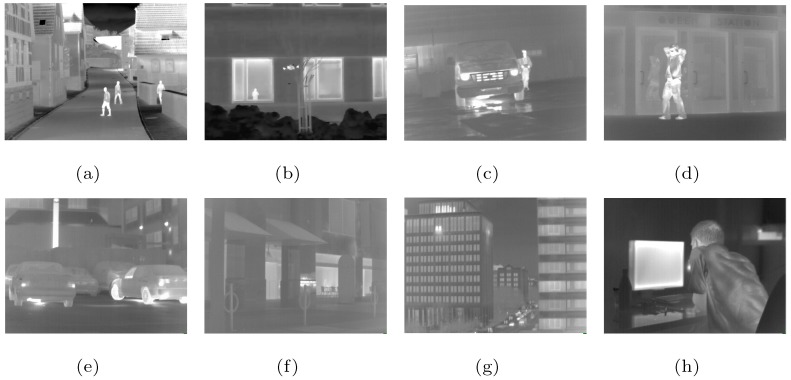
HR infrared images: (**a**) Streets [65], (**b**) Garden [65], (**c**) Station [66], (**d**) Gate [66], (**e**) Cars [66], (**f**) Sidewalk [66], (**g**) Building [66], (**h**) Office [66].

In this section, we present several numerical results to illustrate the performance of the proposed method. RED-HOGS4 is compared with different noise levels and Gaussian blur conditions with several other methods, including the MFT [47], RED-TV [17], RED-TGV [20], and RED-OGSTV [36] methods. Among the four methods, the MFT method used the scripts provided in [47] while other methods are based on the literatures and are combined with the RED framework for super-resolution reconstruction. Eight infrared images are selected from the infrared image database LTIR [65] and IRData [66] as test pictures, as shown in Figure 1. Our experiments were performed on a PC with an Intel CPU 2.8 GHz and 8 GB RAM using MATLAB R2014a.

For the objective evaluation, we calculated the peak signal-to-noise ratio (PSNR) [67] and structural similarity (SSIM) [68]. PSNR is an engineering term, which can compare the similarity of two input images or signals based on the mean square error. SSIM is also a method to measure the similarity between two input images, which is designed to improve on other methods such as PSNR which are not consistent with human eye perception. These can be defined as follows:

(45) $$\begin{array}{cc} \; & \mathrm{PSNR}\left(X,Y\right)=10\times \log_{10}\frac{C_{\max}^{2}}{\sum_{i=0}^{M-1}\sum_{j=0}^{N-1}{\left(X_{ij}-Y_{ij}\right)}^{2}},\\ \; & C_{\max}\leq \left\{\begin{array}{cc} \;\;1, & in\;\;double-precision\;\;intensity\;\;images\\ 255, & in\;\;8-bit\;\;unsigned-integer\;\;intensity\;\;images \end{array}\right. \end{array}$$

(46) $$\begin{array}{c} \mathrm{SSIM}\left(X,Y\right)=\frac{\left(2u_{X}u_{Y}+{\left(255k_{1}\right)}^{2}\right)\left(2\sigma_{\mathrm{XY}}+{\left(255k_{2}\right)}^{2}\right)}{\left(u_{X}^{2}+u_{Y}^{2}+{\left(255k_{1}\right)}^{2}\right)\left(\sigma_{X}^{2}+\sigma_{Y}^{2}+{\left(255k_{2}\right)}^{2}\right)} \end{array}$$

where X and $X_{ij}$ are the original image; Y and $Y_{ij}$ are the reconstructed image; $u_{X}$ and $u_{Y}$ are the mean values of X and Y, respectively. Further, $\sigma_{X}^{2}$ and $\sigma_{Y}^{2}$ are the variances of X and Y, respectively; $\sigma_{\mathrm{XY}}$ is the covariance of X and Y. The parameters $k_{1}$ and $k_{2}$ are set such that the denominator of SSIM is a nonzero number. In this study, we set $k_{1}=0.01$ and $k_{2}=0.03$ [68].

In general, larger values of PSNR and SSIM indicate better performance. Therefore, in this experiment, we focus on the PSNR as well as the SSIM. In all experiments, we set the parameters empirically as follows: μ=1, ρ=0.001, N=3 [57]. If γ=1, the Algorithm 3 is a classic ADMM, but γ=1.618 makes it converge noticeably faster than γ=1 [38]; therefore, we set γ=1.618. Besides, for the tol value in Algorithm 3, when N is recommended to be set to 3 in the literature [57], we found that when tol=0.001, the PSNR value is high, so we set tol=0.001 in all experiments. The blur matrix H in Equation (11) is set as a corresponding matrix to the blur kernel, which was generated by a MATLAB built-in command “fspecial (‘gaussian’, 7, 1.6)”. S is set as a K-fold downsampling operator which is generated by the MATLAB built-in function “downsample(X,K)”.

### 4.2. Infrared Image Super-Resolution Experiment without Noise

In the experiment, the LR images without noise are obtained by downsampling the HR images (2-fold, 3-fold, and 4-fold). To evaluate the performance objectively, PSNR and SSIM are calculated under different levels of super-resolving operators (corresponding to ×2, ×3, and ×4). The experimental results of each method are listed in Table 1.

It can be seen from the experimental results in Table 1 that when there is no noise in the infrared images, in the ×2 reconstruction results, the PSNR values of Street are MFT 31.6188 dB, RED-TV 36.3936 dB, RED-TGV 36.4216 dB, RED-OGSTV 36.3983 dB, and RED-HOGS4 36.4195 dB. The results show that: 1. compared RED-HOGS4 36.4195 dB with RED-OGSTV 36.3983 dB, the high-order has improvement compared with the first-order; 2. compared RED-HOGS4 36.4195 dB with RED-TGV 36.4216 dB, though the result of RED-HOGS4 is worse than that of RED-TGV, the experimental values are close. The SSIM values of Street are MFT 0.9561, RED-TV 0.9867, RED-TGV 0.9911, RED-OGSTV 0.9915, and RED-HOGS4 0.9912. Because the parameter setting in the experiment is mainly to ensure PSNR, not SSIM, the SSIM is given as a reference. However, it can be seen from SSIM results that the SSIM of RED-HOGS4 is only worse than that of RED-OGSTV in five methods. Besides, the best PSNR values of Station, Building and Office are RED-OGSTV 33.1898 dB, RED-TGV 33.6576 dB and RED-TGV 31.2934 dB, while the PSNR values of RED-HOGS4 are 33.1657 dB, 33.6553 dB, 31.2891 dB, respectively. The difference between them is little. On the contrary, we can see the best PSNR values of Garden, Gate, Car and Sidewalk are RED-HOGS4 44.1652 dB, 32.1942 dB, 32.6179 dB and 33.8876 dB in the ×2 reconstruction results. Taken together, although the RED-HOGS4 method only has the best PSNR value for the four images in the ×2 reconstruction results, from an overall perspective, the average PSNR of the eight images processed by the RED-HOGS4 method is greater than that of other methods. The RED-HOGS4 method exhibits better SSIM only in individual pictures; however, the mean SSIM is worse than the processing result obtained by the MFT method. Simultaneously, the PSNR values of all the images processed by the MFT method are poor. As the super-resolution levels of super-resolving operators increase to ×3 and ×4, the results of RED-HOGS4 become significantly better than several other methods. For example, in the ×3 reconstruction results, the PSNR values of RED-HOGS4 are higher than those of RED-OGSTV by about 0.02 dB~0.08 dB. Further, in the ×4 reconstruction results, the deference is expanded to 0.02 dB~0.23 dB. Meanwhile, the SSIM values of RED-HOGS4 are also higher than those of other methods. However, as the RED-HOGS4 method is relatively complex, it takes the longest time compared to all other methods.

**Figure 2:**
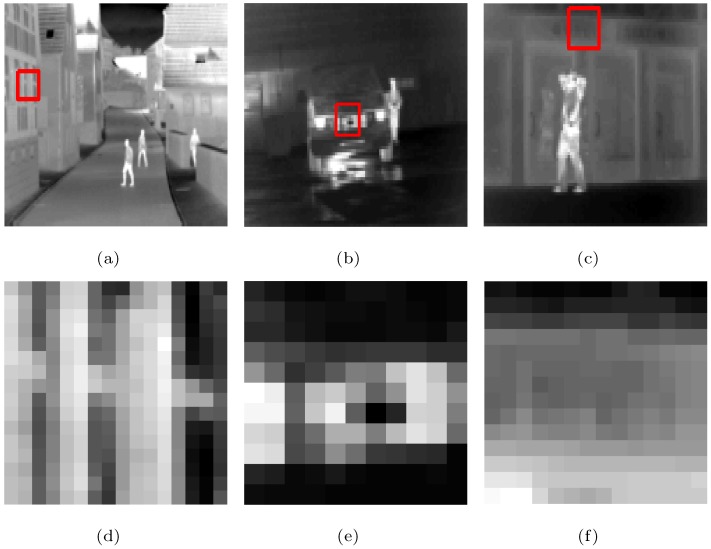
LR infrared images that are down-sampled 4-fold without noise: (**a**) Street, (**b**) Station, (**c**) Gate; (**d**–**f**) enlarged details from the rectangles in (**a**–**c**), respectively.

**Figure 3:**
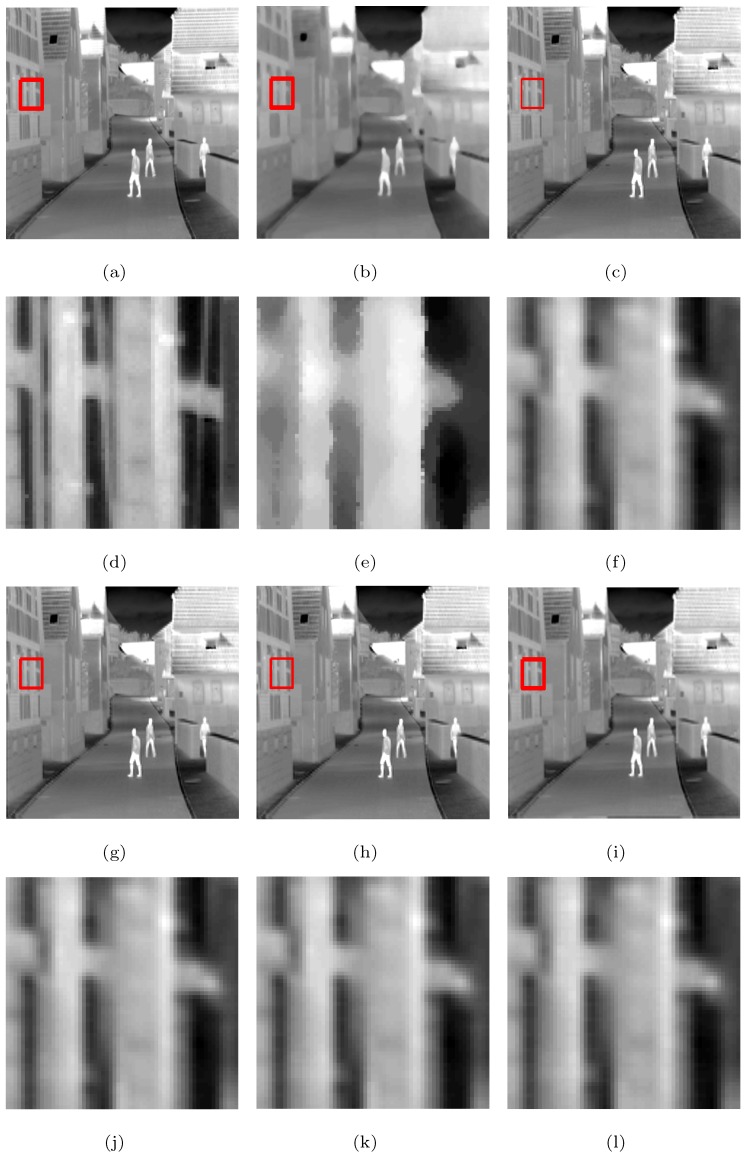
Super-resolution ×4 results of the LR Street images without noise. (**a**–**c**,**g**–**i**) are original image and the results of MFT, RED-TV, RED-TGV, RED-OGSTV, and RED-HOGS4 methods, respectively. (**d**–**f**,**j**–**l**) show the enlarged details from the rectangles in (**a**–**c**,**g**–**i**), respectively.

**Figure 4:**
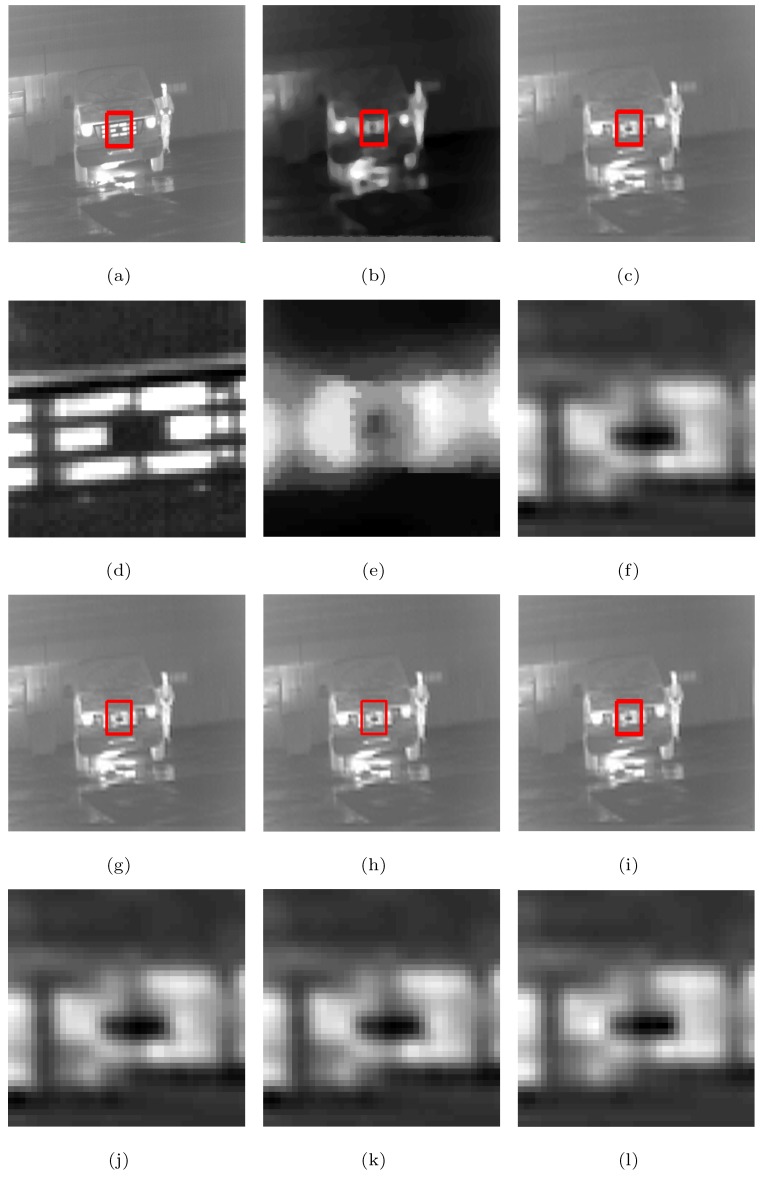
Super-resolution ×4 results of the LR Station images without noise. (**a**–**c**,**g**–**i**) are original image and the results of MFT, RED-TV, RED-TGV, RED-OGSTV, and RED-HOGS4 methods, respectively. (**d**–**f**,**j**–**l**) show the enlarged details from the rectangles in (**a**–**c**,**g**–**i**), respectively.

**Figure 5:**
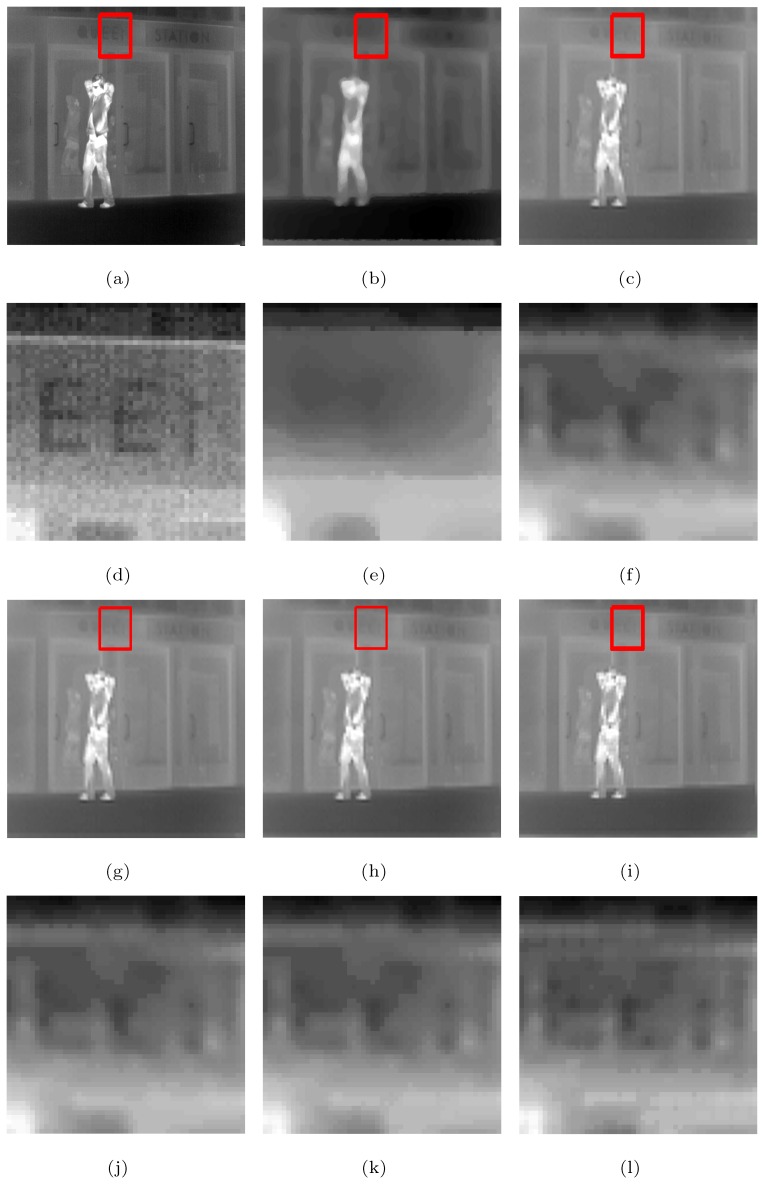
Super-resolution ×4 results of the LR Gate images without noise. (**a**–**c**,**g**–**i**) are original image and the results of MFT, RED-TV, RED-TGV, RED-OGSTV, and RED-HOGS4 methods, respectively. (**d**–**f**,**j**–**l**) show the enlarged details from the rectangles in (**a**–**c**,**g**–**i**), respectively.

The following is a comparison of visual effects on three images: Street, Station, and Gate after 4-fold down-sampling of the original image without any noise using the five methods. The LR images are shown in Figure 2, in which the rectangles are compared with the SRR effects of the five methods in Figure 3, Figure 4 and Figure 5, respectively.

As can be seen from Figure 3, Figure 4 and Figure 5, in the case no noise is introduced, after 4 times super-resolution processing, the effect of MFT processing is the worst among the images obtained by the five methods. The three images obtained by MFT have the phenomenon of unclear boundary and blur. In the visual comparison of images generated through RED-TV, RED-TGV, RED-OGSTV, and RED-HOGS4, we can see that RED-HOGS4 is better for boundary and overall processing of the image. Especially under 4 times magnification in Figure 5, the results of RED-HOGS4 method clearly show the outlines of letters and strokes, which are significantly better than the other methods.

### 4.3. Infrared Image Super-Resolution Experiment with Added White Gaussian Noise

In this experiment, the LR infrared images were generated by downsampling the original images by a factor of two after adding white Gaussian noise of different variance values (σ=5,10,20,30). To evaluate the performance variations based on the noise content for each method objectively, PSNR and SSIM were calculated at the ×2 super-resolving operator. These results are listed in Table 2.

The experimental results show that the MFT method has better PSNR in a few images, but worse PSNR mean values; the processing results of RED-TV and RED-TGV are better than that of the MFT, but worse than those of RED-OGSTV and RED-HOGS4. When the noise is small, the PSNR of the RED-OGSTV method is lower than that of the RED-HOGS4 and its SSIM value is higher than that of RED-HOGS4. With the increase in noise, the reconstruction results of the RED-OGSTV method are further lower than those of the RED-HOGS4 method. In terms of processing time, the RED-HOGS4 is relatively more time consuming compared to the other methods.

**Figure 6:**
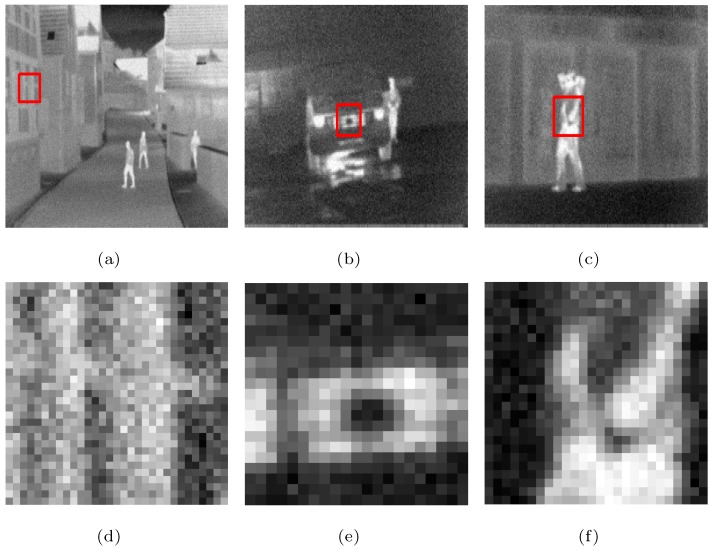
LR infrared images that are downsampled by a factor of two with added white Gaussian noise (σ=10): (**a**) Street, (**b**) Station, (**c**) Gate; (**d**–**f**) enlarged details from the rectangles in (**a**–**c**), respectively.

**Figure 7:**
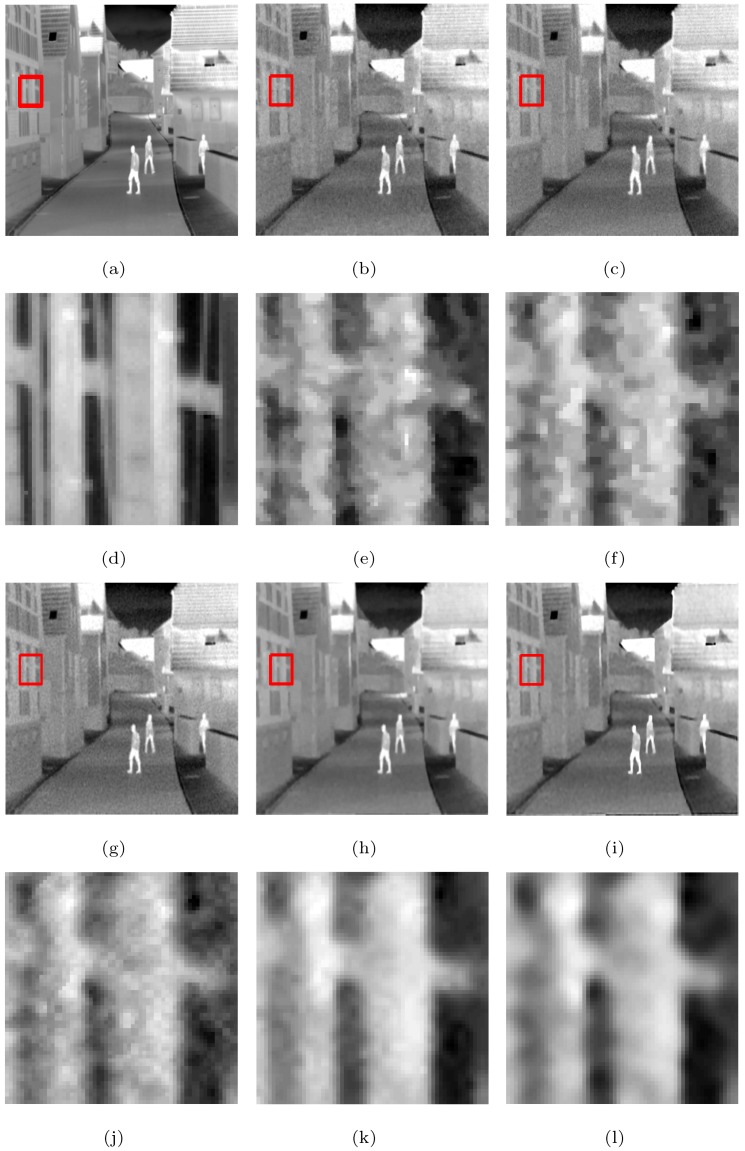
Super-resolution ×2 results of the LR images of the Street with added white Gaussian noise (σ=10): (**a**–**c**,**g**–**i**) original image and the results of the MFT, RED-TV, RED-TGV, RED-OGSTV, and RED-HOGS4 methods, respectively; (**d**–**f**,**j**–**l**) enlarged details from the rectangles in (**a**–**c**,**g**–**i**), respectively.

**Figure 8:**
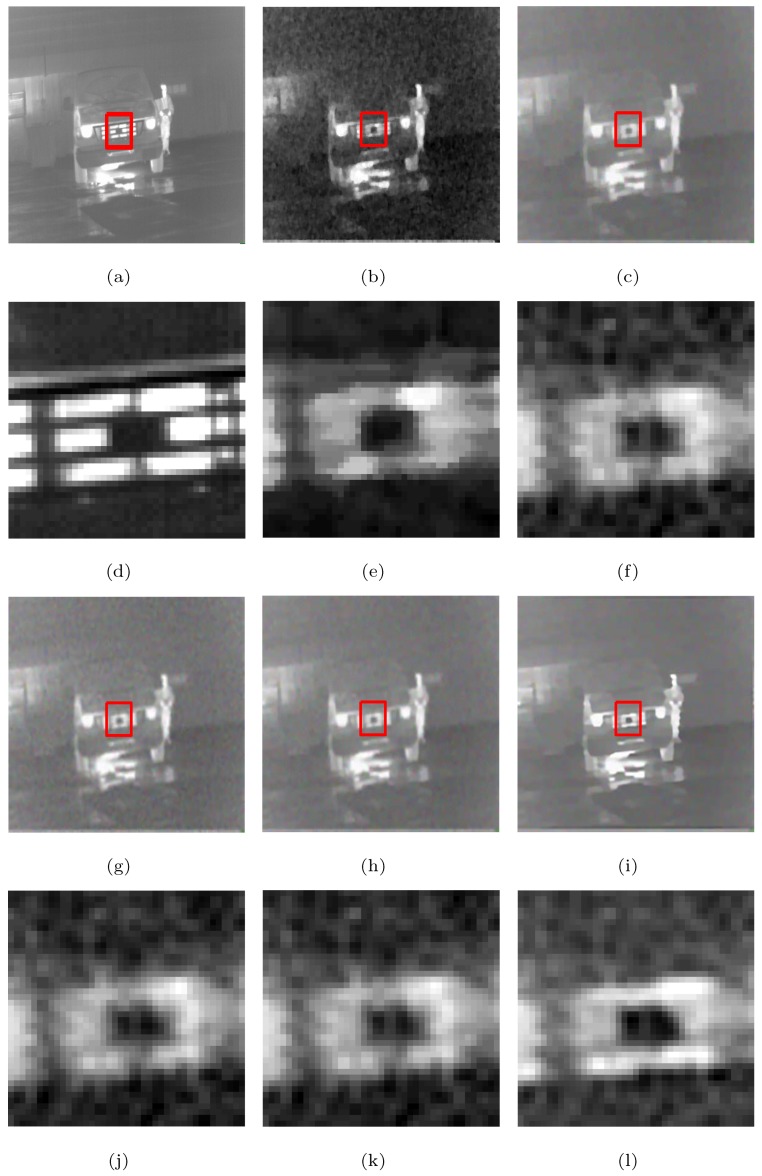
Super-resolution ×2 results of the LR images of the Station with added white Gaussian noise (σ=10): (**a–c**,**g–i**) ground truth and the results of the MFT, RED-TV, RED-TGV, RED-OGSTV, and RED-HOGS4 methods, respectively; (**d**–**f**,**j**–**l**) enlarged details from the rectangles in (**a–c**,**g–i**), respectively.

**Figure 9:**
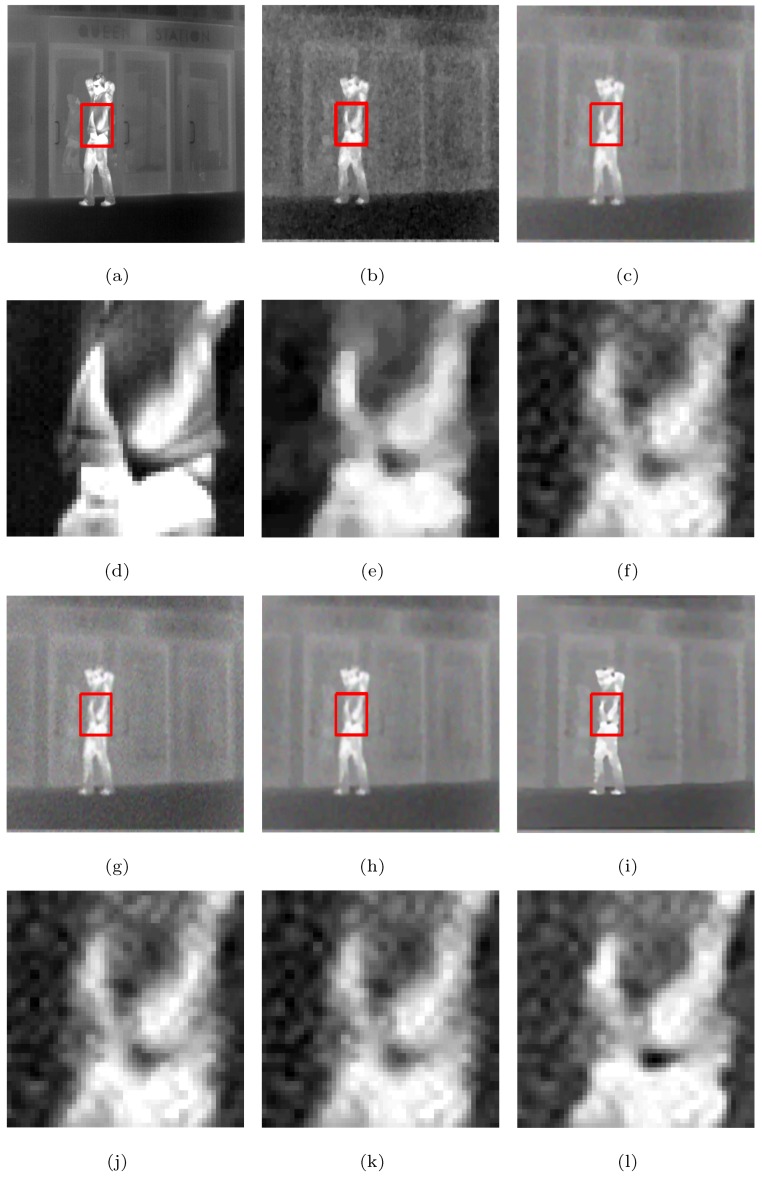
Super-resolution ×2 results of the LR images of the Gate with added white Gaussian noise (σ=10): (**a**–**c**,**g**–**i**) ground truth and the results of the MFT, RED-TV, RED-TGV, RED-OGSTV, and RED-HOGS4 methods, respectively; (**d**–**f**,**j**–**l**) enlarged details from the rectangles in (**a**–**c**,**g**–**i**), respectively.

The visual effects comparison based on the Street, Station, and Gate images, which had added white Gaussian noise (σ=10) and were downsampled by a factor of two, is shown as example in Figure 6, in which the rectangles are compared with the SRR effects of the five methods in Figure 7, Figure 8 and Figure 9, respectively.

From Figure 7, Figure 8 and Figure 9, we see that the MFT has less noise in the reconstructed image, but the entire image is too smooth, resulting in serious loss of boundary information; RED-TV and RED-TGV reconstructed images have inadequate noise removal and information protection, whereas RED-OGSTV and RED-HOGS4 have better reconstructed images, as shown in Figure 7. In Figure 8 and Figure 9, the proposed method shows better results compared to RED-OGSTV for image edge reconstruction and noise effect.

## 5. Discussion

The HOGS4 method adopts quaternion TV and high-order OGSTV, which fully utilizes image correlations in quaternion and extends the first-order overlapping group sparsity to a higher-order such that a clear image can be reconstructed in the presence of noise interference. As to the OGSTV method, in the presence of noise, staircase artifacts are still present, and the noise removal is not as good as that of the HOGS4.

When the MFT method is used to reconstruct an image, regardless of the image being noiseless or noisy, the reconstruction result is very smooth, because of which the details are unclear.

The TV method preserves the edge and detail information of the image and smoothens the image piece by piece; hence, the result usually includes stair artifacts. The TGV method effectively reduces stair artifacts using first-order and second-order gradients during image processing. However, it also causes excessive smoothing and image distortion.

However, compared with other methods, this method is more time consuming because it introduces high-order OGSTV and quaternion, which have higher computational complexity. In the next study, we may use some accelerated iterative methods to improve the convergence speed of the algorithm, thus reducing the time consumption. As the methods in the literatures [26,69], the acceleration operator can be used to reduce the number of iterations of the ADMM algorithm, thereby reducing the time consuming of the super-resolution reconstruction algorithm. Besides, the proposed method may have other shortcomings. For example, the parameter optimization is mainly based on experience; because of the limited number of test infrared images, the parameters may not be fully applied to other sets of infrared images. For practical applications, the parameters are still necessary to optimize for the sets of infrared images. Alternatively, the adaptive mechanism of parameter optimization can be adopted in conjunction with this method.

## 6. Conclusions

In this paper, an infrared image super-resolution reconstruction method based on quaternion overlapping group sparse is proposed. This method produces improved image super-resolution reconstruction capability because it uses a combination of quaternion total variation and high-order group sparse methods. In addition, by introducing the RED framework, the super-resolution problem is transformed into multiple denoising sub-problems. When addressing these sub-problems, multiple difference operators are processed in convolution form. Using this method, according to the convolution theory, it can be converted to frequency domain operations, thereby avoiding large-scale matrix operations. Compared to MFT, TV, TGV, and OGSTV methods, the experimental results prove that the proposed method has better performance.

Although the proposed method only focuses on HOGS4, it can be easily extended to other regular models, such as TGV model, and combined with other methods, such as Lp quasinorm, to improve the performance of super-resolution reconstruction. Besides, in practical application, the method can be used for super-resolution reconstruction or denoising of grayscale images. We will continue to perform these extensions in our follow-up work.

## Figures and Tables

**Table 1:** Infrared image super-resolution experiment results without noise.

Scale	Methods	Street	Garden	Station	Gate	Car	Sidewalk	Building	Office
		PSNR/SSIM/TIME	PSNR/SSIM/TIME	PSNR/SSIM/TIME	PSNR/SSIM/TIME	PSNR/SSIM/TIME	PSNR/SSIM/TIME	PSNR/SSIM/TIME	PSNR/SSIM/TIME
×2	MFT	31.6188/0.9561/14.96	38.2022/0.9898/14.18	29.4866/**0.9250**/3.14	29.6519/**0.9273**/3.12	29.5091/**0.9201**/3.24	31.3036/**0.9350**/3.26	29.9346/0.8781/3.1	28.6855/**0.9208**/3.1
	TV	36.3936/0.9867/4.41	43.6264/0.9934/5.35	32.6511/0.8780/1.86	31.7051/0.8672/1.54	32.1085/0.8719/1.4	33.2392/0.8767/1.36	33.3386/0.8997/1.53	31.0775/0.8688/1.79
	TGV	**36.4216**/0.9911/11.45	43.8369/0.9943/11.9	33.0034/0.9051/3.79	31.9684/0.8855/3.48	32.4560/0.8984/3.31	33.5317/0.8974/3.63	**33.6576**/0.9152/3.31	**31.2934**/0.8891/3.28
	OGSTV	36.3983/**0.9915**/17.52	44.1539/**0.9965**/19.38	**33.1898**/0.9236/3.14	32.1813/0.9129/3.17	32.6052/0.9152/2.65	33.8642/0.9223/3.56	33.6569/0.9153/3.79	31.2804/0.8867/3.48
	HOGS4	36.4195/0.9912/18.77	**44.1652**/0.9964/20.71	33.1657/0.9222/4.38	**32.1942**/0.9141/3.83	**32.6179**/0.9153/3.36	**33.8876**/0.9225/3.91	33.6553/**0.9155**/4.36	31.2891/0.8891/3.45
×3	MFT	28.0181/0.8737/8.38	33.6661/0.9639/8.46	27.4429/0.8878/2.32	28.0566/0.8947/2.03	27.2375/0.8780/2.07	29.5699/0.9004/1.95	27.4554/0.7737/2.01	26.5407/0.8804/2.06
	TV	33.4016/0.9405/4.71	41.2186/0.9932/6.46	30.7039/0.9256/1.81	30.2514/0.9246/1.51	30.6114/0.9237/1.62	32.0579/0.9326/1.68	31.9141/0.9202/1.5	29.2032/0.9241/1.47
	TGV	33.4061/0.9587/13.92	41.3028/0.9918/13.67	30.5973/0.9303/3.34	30.144/0.9250/3.43	30.5905/0.9024/3.42	32.0108/0.9236/3.57	31.9015/0.9223/3.39	29.4769/0.9239/3.49
	OGSTV	33.4173/0.9653/16.69	41.3054/0.9902/19.03	30.6362/**0.9337**/2.87	30.2387/0.9263/3.28	30.6197/0.9252/2.56	32.0310/0.9218/3.29	31.9027/0.9233/3.26	29.4788/0.9276/3.32
	HOGS4	**33.4604**/**0.9748**/17.99	**41.3245**/**0.9940**/20.09	**30.7198**/0.9336/3.19	**30.2643**/**0.9350**/4.04	**30.6401**/**0.9311**/3.13	**32.1018**/**0.9475**/3.9	**31.9334**/**0.9279**/4.16	**29.5196**/**0.9381**/3.46
×4	MFT	26.2322/0.8100/5.96	31.1897/0.9345/5.73	26.2479/0.8604/1.54	27.0864/0.8727/1.59	25.8728/0.8460/1.53	28.6704/0.8795/1.54	26.2847/0.7174/1.54	25.1938/0.8457/1.5
	TV	30.6168/0.9114/3.79	37.8652/0.9842/5.15	29.2303/0.9156/1.73	29.0929/0.9279/1.67	29.2363/0.9158/1.65	30.8118/0.9301/1.53	29.9565/0.8716/1.28	28.0533/0.9035/1.84
	TGV	30.5942/0.9082/11.17	37.8796/0.9845/11.51	29.2475/0.9142/3.39	29.2905/0.9301/3.56	29.6773/0.9149/3.48	30.8159/0.9203/3.18	29.9634/0.8562/0.62	28.1761/0.9067/3.51
	OGSTV	30.6149/0.9108/15.77	37.8722/0.9818/19.8	29.2319/0.9188/3.00	29.3264/0.9249/3.38	29.7456/0.9086/6.75	30.8085/0.9212/3.73	29.9470/0.8717/2.93	28.1810/0.9072/3.42
	HOGS4	**30.8434**/**0.9374**/16.27	**37.9156**/**0.9868**/21.73	**29.3724**/**0.9229**/3.43	**29.5356**/**0.9335**/4.04	**29.8813**/**0.9186**/6.86	**30.8252**/**0.9316**/3.96	**30.0449**/**0.8750**/3.73	**28.2137**/**0.9202**/4.41

**Table 2:** Infrared image super-resolution ×2 experiment results with added white Gaussian noise.

σ	Methods	Street	Garden	Station	Gate	Car	Sidewalk	Building	Office
		PSNR/SSIM/TIME	PSNR/SSIM/TIME	PSNR/SSIM/TIME	PSNR/SSIM/TIME	PSNR/SSIM/TIME	PSNR/SSIM/TIME	PSNR/SSIM/TIME	PSNR/SSIM/TIME
5	MFT	**30.3844**/0.8947/14.13	35.2944/0.9278/13.71	29.2544/0.8971/3.39	29.3962/0.8971/3.28	29.2663/0.8918/3.29	30.9504/0.9009/3.26	29.7203/0.8560/3.2	28.4523/0.8887/3.28
	TV	30.1995/0.8673/3.56	35.7295/0.9063/3.81	30.5913/0.9213/3.79	30.7728/0.9236/3.39	31.1923/0.9132/1.28	32.0916/0.9291/5.1	30.7949/0.8729/5.82	29.1854/0.9176/9.38
	TGV	30.2973/0.8299/11.58	35.8874/0.9111/11.12	30.5961/0.9143/3.59	30.7538/0.9225/3.71	31.2238/0.9126/3.23	32.1587/0.8649/3.48	30.8175/0.8664/3.43	29.2051/0.9162/3.63
	OGSTV	30.3232/**0.9044**/15.27	35.9191/0.9481/15.58	30.6232/**0.9223**/3.63	30.8097/**0.9252**/2.98	31.2574/0.9044/2.53	**32.1766**/0.9307/3.51	30.8574/0.8755/3.68	29.2161/**0.9212**/7.53
	HOGS4	30.2752/0.8793/22.49	**35.9576**/**0.9511**/19.57	**30.6954**/0.9184/9.44	**30.9303**/0.9201/6.41	**31.3745**/**0.9196**/9.06	32.1300/**0.9317**/6.21	**30.9188**/**0.9003**/7.83	**29.2294**/0.9211/11.87
10	MFT	28.8193/0.8069/14.16	33.6306/0.8761/13.71	28.6383/0.8353/3.46	28.7479/0.8295/3.26	28.5947/0.8278/3.31	29.9312/0.823/3.26	28.9476/0.8024/3.23	27.8191/0.8149/3.37
	TV	28.5648/0.7735/3.95	34.0973/0.9009/8.24	29.8220/0.9056/4.17	30.2015/0.9033/1.92	30.2408/0.8963/1.9	31.4895/0.9131/3.14	29.6852/0.8180/3.67	28.4762/0.8996/8
	TGV	29.0636/0.8046/12.32	34.1716/0.9454/11.73	30.0730/0.8939/3.4	30.1833/0.8575/3.74	30.3142/0.8919/3.21	31.5726/0.8840/3.56	29.8594/0.8207/3.48	28.5003/0.9003/3.53
	OGSTV	29.0669/0.8574/19.8	34.2232/0.9440/16.86	30.0650/0.9050/2.75	30.3300/0.9090/2.43	30.3722/0.9026/2.45	31.6164/0.9171/3.26	29.8875/0.8262/3.1	28.4669/0.9050/6.93
	HOGS4	**29.1334**/**0.8582**/22.12	**34.2920**/**0.9496**/20.45	**30.1622**/**0.9153**/8.59	**30.4083**/**0.9114**/8.98	**30.7266**/**0.9108**/9.99	**31.6563**/**0.9219**/9.04	**29.9636**/**0.8671**/8.45	**28.5119**/**0.9093**/12.27
20	MFT	27.2470/0.7125/14.07	29.1815/0.6796/13.68	26.8062/0.6751/3.23	26.8204/0.6589/3.32	26.6719/0.6656/3.35	27.32540/0.6330/3.28	26.9133/0.6677/3.2	25.9781/0.6361/3.49
	TV	27.2404/0.7474/10.14	31.0762/0.8125/12	28.7096/0.8539/4.7	29.1721/0.8591/2.46	29.1275/0.8520/2.71	30.3201/0.8641/4.27	28.2517/0.7432/4.07	27.7072/0.8620/7.55
	TGV	27.3241/0.7799/11.62	31.5214/0.8712/11.64	28.8323/0.8381/3.45	29.2118/0.8427/3.28	29.1635/0.8699/3.4	30.4717/0.8869/3.6	28.3606/0.7062/3.49	27.8472/0.7391/3.24
	OGSTV	27.3548/0.7504/22.15	31.3022/0.8387/16.52	28.9738/0.8598/2.82	29.3451/0.8651/2.73	29.2848/0.8592/2.92	30.5138/0.8687/2.93	28.3291/0.7002/2.95	27.8163/0.7321/3.42
	HOGS4	**27.7175**/**0.7979**/21.14	**31.8839**/**0.8968**/21.09	**29.3417**/**0.8926**/9.07	**29.6127**/**0.8831**/9.95	**29.8622**/**0.8890**/11.91	**30.7887**/**0.8993**/10.02	**28.5532**/**0.7851**/11.89	**27.8925**/**0.8836**/11.48
30	MFT	24.9228/0.5721/14.41	26.1154/0.516/13.68	24.8624/0.5289/3.29	24.7843/0.5070/3.31	24.6631/0.5187/3.28	24.9161/0.4727/3.31	24.8941/0.5420/3.32	24.0533/0.4860/3.46
	TV	26.0036/0.6568/9.48	28.7370/0.6810/9.33	28.0216/0.7971/3.42	28.3876/0.8006/3.53	28.2183/0.7961/3.14	29.4937/0.8061/3.37	27.4704/0.6891/4.37	27.1226/0.8013/6.12
	TGV	26.1381/0.7028/11.26	29.5419/0.7842/11.72	27.8666/0.7898/3.71	28.2098/0.7820/3.45	28.3737/0.8541/3.48	29.6464/0.8514/3.76	27.5328/0.7341/3.43	27.2024/0.7665/3.49
	OGSTV	26.1238/0.6547/14.65	29.5298/0.8103/19.36	28.2897/0.8634/2.96	28.7690/0.8678/3.43	28.5982/0.8556/3.51	29.8894/0.8450/3.74	27.5448/0.7378/4.32	27.3762/0.8566/5.05
	HOGS4	**26.7282**/**0.7440**/18.03	**29.9149**/**0.8372**/23.99	**28.6433**/**0.8727**/11.37	**28.8957**/**0.8681**/9.66	**29.1366**/**0.8653**/11.72	**29.9323**/**0.8742**/11.73	**27.7340**/**0.7512**/11.58	**27.4272**/**0.8629**/11.06
